# THE ROLE OF REPRODUCTIVE AGING FOR ALZHEIMER’S DISEASE AND RELATED DEMENTIAS IN THE CARDIOVASCULAR HEALTH STUDY

**DOI:** 10.1093/geroni/igad104.1441

**Published:** 2023-12-21

**Authors:** Alexis Reeves, Oscar Lopez, Michelle Shardell, Michelle Odden

**Affiliations:** Stanford University; School of Medicine, Stanford, California, United States; University of Pittsburgh, Pittsburgh, Pennsylvania, United States; University of Maryland School of Medicine, Baltimore, Maryland, United States; Stanford University, Stanford, California, United States

## Abstract

Most US individuals with ADRD are women. As early as midlife, Black individuals have nearly double the ADRD risk than Whites. Menopause occurs in midlife, accompanied by declines in potentially neuro-protective and cardio-protective hormones. Earlier menopause possibly means earlier exposure to decreased sex hormones, which increases ADRD risk. Surgical menopause via reproductive surgeries causes a sudden loss of hormones and could be a modifiable ADRD risk factor. We used data from 1,977 women prospectively followed for an average of 7.5 years, to determine if menopausal type (natural/surgical) and pre-mature menopause (<40 years) are associated with adjudicated-ADRD. Participants self-reported age of natural menopause and/or surgery (hysterectomy and/or bilateral-oophorectomy), hormone-therapy, education, and race (Black/White). Adjudication for ADRD was conducted by neurologist/psychiatrist committee review using neuropsychological tests, neurological examinations, medical records, physician questionnaires, and proxy/informant interviews. Mean age was 78, 15% were Black, 25% had less than a high-school education, 18% had ADRD, 13% had pre-mature menopause, and 42% surgical menopause. Black women had higher prevalence of pre-mature (22% versus 12%) and surgical (54% versus 29%) menopause versus White women. Pre-mature menopause was associated with a non-significant higher hormone use-adjusted hazard of ADRD (HR=1.09 [0.95-1.25]), findings were attenuated after adjustment for education (HR=1.08 [0.94-1.24]) and race (HR=1.01 [0.88-1.17]). Surgical menopause was associated with higher hormone use-adjusted hazard of ADRD (HR=1.12 [1.02-1.24]), findings were similar after adjustment for education (HR=1.12 [1.01-1.23]) and attenuated after adjustment for race (HR=1.03 [0.93-1.14]). Further work examining menopause, ADRD, and contributions to Black-White disparities are warranted.

